# Severity and properties of cardiac damage caused by *Streptococcus pneumoniae* are strain dependent

**DOI:** 10.1371/journal.pone.0204032

**Published:** 2018-09-14

**Authors:** Anukul T. Shenoy, Sarah M. Beno, Terry Brissac, Jeremiah W. Bell, Lea Novak, Carlos J. Orihuela

**Affiliations:** 1 Department of Microbiology, The University of Alabama at Birmingham, Birmingham, Alabama, United States of America; 2 Department of Pathology, The University of Alabama at Birmingham, Birmingham, Alabama, United States of America; University of Mississippi Medical Center, UNITED STATES

## Abstract

*Streptococcus pneumoniae* is an opportunistic Gram-positive pathogen that can cause invasive disease. Recent studies have shown that *S*. *pneumoniae* is able to invade the myocardium and kill cardiomyocytes, with one-in-five adults hospitalized for pneumococcal pneumonia having a pneumonia-associated adverse cardiac event. Furthermore, clinical reports have shown up to a 10-year increased risk of adverse cardiac events in patients formerly hospitalized for pneumococcal bacteremia. In this study, we investigated the ability of nine *S*. *pneumoniae* clinical isolates, representing eight unique serotypes, to cause cardiac damage in a mouse model of invasive disease. Following intraperitoneal challenge of C57BL/6 mice, four of these strains (D39, WU2, TIGR4, and 6A-10) caused high-grade bacteremia, while CDC7F:2617–97 and AMQ16 caused mid- and low-grade bacteremia, respectively. Three strains did not cause any discernible disease. Of note, only the strains capable of high-grade bacteremia caused cardiac damage, as inferred by serum levels of cardiac troponin-I. This link between bacteremia and heart damage was further corroborated by Hematoxylin & Eosin and Trichrome staining which showed cardiac cytotoxicity only in D39, WU2, TIGR4, and 6A-10 infected mice. Finally, hearts infected with these strains showed varying histopathological characteristics, such as differential lesion formation and myocytolysis, suggesting that the mechanism of heart damage varied between strains.

## Introduction

*Streptococcus pneumoniae* (the pneumococcus) is a Gram-positive bacteria and common resident of the human nasopharynx. It is also an opportunistic pathogen that can cause many different types of disease including otitis media, pneumonia, bacteremia, and meningitis [1]. These infections primarily occur in the very young and old, as their immune systems are under-developed or waning, respectively [2–4]. While the overall attack rate of the pneumococcus is low, so many individuals are colonized that the pneumococcus is among the leading causes of infectious deaths worldwide [5, 6]. The World Health Organization estimates that 500,000 children under five years of age die each year from pneumococcal disease [7]. Furthermore, the overall mortality associated with invasive pneumococcal disease (IPD) in adults increases from 1.53/100,000 at 50 years of age to 11.56/100,000 at 85+ [8].

A link between pneumonia and acute cardiac events has been known since the 1930s [9–11]. Several recent clinical studies by Corrales-Medina have revived an appreciation for this serious complication of pneumonia [12, 13]. Relevant to our study, Musher et al. showed that up to 20% of adults hospitalized for pneumococcal pneumonia experienced congestive heart failure, arrhythmia, and myocardial infarction, either alone or in combination [14]. These individuals were up to fourfold more likely to die than those hospitalized for pneumococcal pneumonia without a cardiac complication [14]. Even more recently, Eurich et al. showed that pneumococcal bacteremia is a risk-factor for adverse cardiac events in convalescence for up to 10 years [15]. Thus, some form of acute and long-lasting cardiac damage is incurred during severe pneumonia that results in invasive pneumococcal disease (IPD).

Helping to explain why this occurs, our laboratory has recently demonstrated that *S*. *pneumoniae* is capable of translocation across the vascular endothelium and invasion of the myocardium from the bloodstream. Within the heart, *S*. *pneumoniae* kill cardiomyocytes and resident and infiltrating macrophages, causing extensive myocardial damage [16–19]. At sublethal levels, pneumococcal cell wall and pneumolysin, the *S*. *pneumoniae* pore-forming toxin, also impair cardiac contractility [20, 21]. The former is due to cell wall ligation of platelet-activating factor receptor and toll-like receptor 2 which can activate NFkB; NFkB activation is inhibitory to cardiac contractility [22]. The latter the result of pneumolysin-mediated membrane damage which disrupts the calcium signaling that is essential for muscle contraction [21]. Together, these and the physiological stressors that are present during severe infection result in cardiac dysfunction and heart failure.

Importantly, the majority of work on *S*. *pneumoniae* invasion *in vivo* using the mouse model has been done using two strains: serotype 4 strain TIGR4 and serotype 2 strain D39. In the heart, TIGR4 actively replicates to form bacteria-filled lesions, which we have called cardiac microlesions. TIGR4 in microlesions have salient biofilm properties, such as intrinsic resistance to antimicrobials [19]. In contrast, strain D39 does not form these lesions; nonetheless, D39 is associated with considerable tissue damage and the infiltration of immune cells at sites where the bacteria are present [17, 21]. These observations suggest that different pneumococcal strains incite different and yet unknown cardiopathologies. This possibility is especially pertinent since clinical isolates of *S*. *pneumoniae* vary by as much as 10% of their genomic content from each other [23], and this estimate does not include the variability in expression or encoded primary amino acid sequence for shared *S*. *pneumoniae* genes [19, 24–26].

Moving forward, obtaining an understanding the full range of pathology associated with different pneumococcal serotypes relevant to pneumonia-associated adverse cardiac events (PACE) may be useful for determining prophylactic and therapeutic options for prevention and/or treatment. Here, we report a survey of nine different *S*. *pneumoniae* clinical isolates belonging to eight distinct serotypes in mice. Our goal was to take advantage of the strength of the mouse model to identify the cardiopathology observed during IPD and identify common aspects that were shared between cardiotoxic strains.

## Materials and methods

### Bacterial strains

Strains of *S*. *pneumoniae* used in this study and their capsular serotypes are listed in Table 1. For all experiments, *S*. *pneumoniae* were grown in Todd Hewitt Broth (Acumedia, Neogen) supplemented with 0.5% yeast extract (THY) at 37°C in 5% CO_2_. Bacteria used were in exponential phase of growth and taken when broth cultures reached an optical density of OD_620_ = 0.35. This corresponded to approximately 1x10^8^ CFU/mL. Isolate source details are available in S1 Table.

**Table 1:** *S*. *pneumoniae* strains used throughout this study.

Strain	Capsular Serotype	Reference
D39	2	[27]
WU2	3	[28]
TIGR4	4	[29]
AMQ16	5	This study
6A-10	6A	[30]
CDC7F:2617–97	7F	This study
EF3030	19F	[31]
BHN97	19F	[32]
CDC23F:2216–94	23F	[33]

### Ethics statement

All mouse experiments were reviewed and approved by the Institutional Animal Care and Use Committees at The University of Alabama at Birmingham, UAB (Protocol # IACUC-20175). All animal care and experimental protocols adhered to Public Law 89–544 (Animal Welfare Act) and its amendments, Public Health Services guidelines, and the Guide for the Care and Use of Laboratory Animals (U.S. Department of Health & Human Services). Mice were deemed moribund and were euthanized when they exhibited trembling, a ruffled fur coat, a hunched position, and demonstrated severe immobility upon manual contact. Following challenge of mice, standard chow soaked in water was placed within each cage so as to help prevent dehydration.

### Infection of mice

Female 6 to 8-week-old C57BL/6J mice (The Jackson Laboratory) were challenged by intraperitoneal (*i*.*p*.) injection with 10^3^ CFU of exponential phase pneumococci in 100 μL phosphate-buffered saline (PBS). Six mice were challenged per strain. We previously determined that this challenge route recapitulates the cardiac damage that occurs as result of pneumonia with bacteremia, but is more amendable to uniform disease severity [34] and therefore reduces the total number of animals needed. Blood for assessment of bacterial burden was obtained by tail snip and collection of 2 μL of blood. Peripheral blood for serum samples was collected by retro-orbital bleeding of anesthetized mice just prior to euthanasia. At fixed time points, or when deemed moribund, mice were euthanized by isoflurane asphyxiation. Death was confirmed by opening of chest cavity during extraction of the heart. Mice were perfused with sterile PBS by puncturing the left ventricle with a syringe and snipping of the right atrium to remove blood-circulating bacteria from the coronary vasculature before processing the hearts. Strain CDC7F:2617–97, was additionally tested using *i*.*p*. challenge at a higher inoculum (10^5^ CFU) in five mice. To determine whether non-invasive strains could cause cardiac damage in a more susceptible host, i.e. allow higher bacterial burden in the blood, an additional set of mice (n = 5) were experimentally depleted of neutrophils by *i*.*p*. injection of 100 μg anti-Ly6G antibody (BioXCell, clone RB6-8C5) 24 hours before challenge with 10^3^ CFU of CDC7F:2617–97 [35]. These mice were euthanized 26 hours post-infection. Importantly, monoclonal antibody from the RB6-8C5 clone is specific to the Gr1 complex, which includes Ly6G, which is present on neutrophils, and Ly6C, which is found on macrophages and dendritic cells.

For all mouse experiments, mice were monitored for health and behavior at least three times per day (early morning, mid-day, and early evening). There were no unexpected mouse deaths. Mice were challenged mid-day so that they could be carefully observed during the course of infection. All mice were housed in their home cages throughout these experiments. Of note, mice were randomly assigned to challenge groups.

### Assessment of bacterial burden in the heart or blood

Pneumococcal burden in the blood of the mice was determined by performing serial dilution of blood in PBS, plating on blood agar, and extrapolating from the colony counts the next day. Bacterial burden in the hearts of the same mice was determined by plating serial dilutions of homogenized hearts. Briefly, excised hearts were homogenized in pre-weighed tubes containing 1 mL of sterile PBS, allowing for weighing and normalizing to organ volume. The bacterial titers were then extrapolated from colony counts after overnight incubation. Plates were incubated at 37°C in 5% CO_2_ and burden was expressed as CFU/mL of blood or CFU/g of tissue.

### Strain survival in human serum

*S*. *pneumoniae* were tested for their ability to survive in human serum (Sigma Aldrich, H4522). Briefly, bacteria were grown in THY broth to an OD_620_ of 0.35, diluted in 100 μL, and combined with 900 μL of human serum for a starting concentration of 10^4^ CFU/mL. Inoculated serum was incubated at 37°C and from which samples were taken and plated on Blood Agar every 15 minutes for two hours. Each strain was tested in duplicate and data was presented as a percentage of survival.

### Measurement of cardiac troponin-I (cTn-I) levels in the serum

Retro-orbital bleeds of anesthetized mice were collected in 1.5 mL tubes and centrifuged at 300 *xg* for 10 minutes. The transparent serum layer above the RBC pellet was removed and stored as single-use 100 μL aliquots at -80°C. Levels of cardiac troponin-I in the serum were measured by high-sensitivity ELISA using manufacturer’s protocol (Life Diagnostics, West Chester, PA; cat # CTNI-1-HS).

### Processing and staining of cardiac tissue

Perfused hearts collected from euthanized mice were washed thoroughly with PBS. One portion of the heart tissue was embedded in cassettes with Optimal Cutting Temperature Compound (Tissue-Tek, 4583) for immunofluorescent staining. Frozen 8 μm thick cardiac sections were fixed with 10% neutral buffered formalin, permeabilized in 0.2% Triton X and blocked with PBS containing 5% serum from species to which the secondary antibody belonged (blocking buffer). Sections were then incubated overnight at 4°C with blocking buffer containing a 1:1000 dilution of primary antibody: rabbit anti-sera specific for the serotype used for infection (Statens Serum Institut). The next day, sections were vigorously washed with 0.2% Triton X and then incubated for 1 hour at room temperature with blocking buffer containing secondary antibody at 1:2000 dilution: FITC labeled goat α-rabbit antibody (Jackson Immuno Research, cat #111-096-144), or Rhodamine (TRITC) labeled donkey α-rabbit antibody (Jackson Immuno Research, cat #711-025-152). Sections were stained for TUNEL using DeadEnd^TM^ Fluorometric TUNEL system (Promega, G3250) using the manufacturer’s protocols. All slides were stained with DAPI (Molecular Probes by Life Technologies, R37606) and sections were mounted with FluorSave (Calbiochem: 345789).

For histology, the remaining portions of heart tissue were processed and embedded in paraffin. From these blocks, 5 μm thick sections were cut and stained using Hematoxin & Eosin (H&E) at the UAB Pathology Core. Trichrome staining of the paraffin embedded sections was also performed using manufacturer’s protocols (Abcam, cat # ab150686).

### Monitoring of pneumolysin, choline binding protein A, and H_2_O_2_ production

Bacterial expression of pneumolysin (Ply) and choline-binding protein A (CbpA) were assessed by immunoblot. Briefly, mid-log phase cultures of *S*. *pneumoniae* were pelleted by centrifugation, washed in PBS and lysed by addition of pneumococcal lysis buffer (0.01% sodium dodecyl sulfate, 0.1% deoxycholate, and 0.015 M sodium citrate). Protease inhibitors were added to these lysates. Protein concentration was determined using the Bicinchoninic Acid assay kit (Sigma-Aldrich cat # BCA1) according to the manufacturer’s protocol. On a 10% polyacrylamide gel (Bio-Rad cat # 161–0183), 5 μg of protein were loaded and separated before transfer onto a nitrocellulose membrane (Bio-Rad cat #170–4270). Membranes were blocked in 5% Non-fat dry milk before incubation with anti-pneumolysin (1/1000e, abcam, ab71811) or anti-CbpA (1/1000) [36] for 1 hour at room temperature. Membrane were then washed in TBS-0.1%Tween20 and incubated with horse radish peroxidase-conjugated goat anti-rabbit (1/10000e, Jackson). Membrane were washed and signal was detected using Clarity™ Western ECL and ChemiDoc XRS+ (Bio-Rad cat #170–5061). H_2_O_2_ production was assessed for each strain using the Pierce^TM^ Quantitative Peroxide Kit- Aqueous (Thermo Scientific, cat # 23280) according to manufacturer’s protocols.

### Statistical analysis

Statistical comparisons between cohorts at a single measured time point were done using a non-parametric one-way ANOVA. Linear regression and Pearson’s Correlation analyses were performed for correlation studies. All statistical analyses were executed using GraphPad Prism 7.0 (GraphPad Software Inc, La Jolla, CA). Data are represented as mean ± SEM. *P*-values ≤ 0.05 were deemed significant.

## Results

### Bacterial burden in heart and serum levels of cardiac troponin are positively correlated to bacterial burden in blood across diverse strains

**Fig 1:**
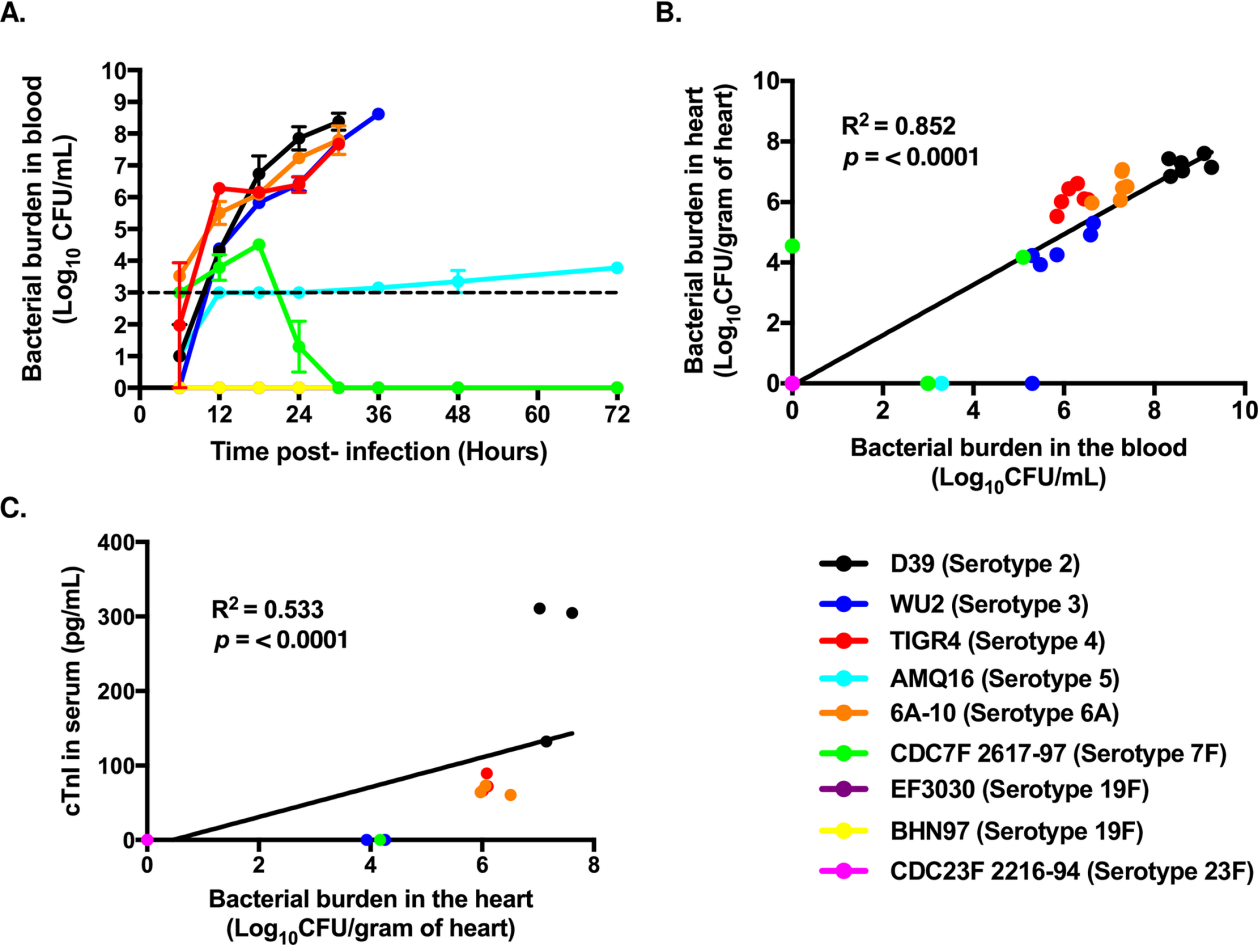
High-grade bacteremia is prerequisite to myocardial invasion and myocardial damage. **A)** Bacterial burden of different strains of pneumococci circulating in the blood at designated time points following *i*.*p*. challenge of mice (n = 6 mice per strain). Points represent the average titer for the mice infected with each strain. Dashed line indicates limit of detection. **B)** Linear Regression analysis of pneumococcal burden recovered from the heart versus the pneumococcal burden in the blood of the same mice (n = 6 mice per strain). **C**) Linear Regression analysis of detectable troponin-I (cTn-I) in the blood of infected mice correlated versus pneumococcal burden recovered from the heart of the same mice (n = 3 mice per strain). Note, the limit of detection for the cTn-I ELISA is 0.156 ng/mL. Data are represented as mean ± SEM.

Mice were challenged *i*.*p*. with 10^3^ CFU of one of the nine different pneumococcal isolates listed in Table 1. Prior to challenge, all mice exhibited normal behavior. Subsequently, at 30 hours post-infection, we assessed the prevalence and severity of bacteremia, the number of pneumococci that had invaded the heart, and levels of cardiac troponin-I, a marker of cardiac injury, in serum. Surprisingly, only four of the clinical isolates tested were capable of causing high-grade bacteremia (greater than 10^5^ CFU/mL of blood): D39, WU2, TIGR4, and 6A-10 (Fig 1A). EF3030, BHN97, and CDC23F:2216–94 were undetected in blood samples at all time points tested. AMQ16 caused sustained but low-grade bacteremia, with individual mice alternating between detectable and undetectable counts in successive time points. Finally, CDC7F:2617–97 was initially capable of mid-grade bacteremia but was cleared from the bloodstream by 16 hours post-infection. Note, that our threshold for detection in this instance was 10^3^ CFU/mL of blood. Critically, the number of pneumococci present in the blood of mice at 30 hours post-infection was strongly and positively correlated (R^2^ = 0.852; *P* < 0.0001) with the number of pneumococci recovered from perfused and homogenized heart samples from the same animals (Fig 1B). In turn, there was a weaker but still positive correlation between levels of bacteria in the heart and serum levels of troponin-I (Fig 1C). Thus, the ability of *S*. *pneumoniae* to invade the heart and trigger the release of troponin seemed to be dependent upon the number of bacteria in the bloodstream. To validate this, hearts from infected mice were sectioned and invaded pneumococci visualized by immunofluorescence against capsule (S1 Fig). Only mice with high-levels of bacteremia showed bacteria in the heart.

We sought to assess if increased bacteremia alone would enhance the capacity of CDC7F:2617–97 to cause cardiac damage. Mice infected with a 100-fold higher dose (10^5^ CFU) of CDC7F:2617–97 also developed bacteremia, however it was not more severe than mice that had been challenged with 10^3^ CFU (*P =* 0.2251). The average blood titer at euthanasia was 2.48x10^3^ CFU/mL (S2 Fig). Whereas only two of six (33.3%) mice challenged with CDC7F:2617–97 at 10^3^ CFU had bacteria in the heart at time of euthanasia, three of five (60%) mice challenged with 10^5^ CFU had detectable bacteria in the heart. Thus, there was a modest increase in cardiac involvement. However, the difference in bacterial burden in heart was not significant (*P* = 0.8701). Pre-treatment of mice with antibody against Ly6G, which depletes neutrophils, and to lesser extent monocytes and macrophages [35], did not enhance disease severity for CDC7F:2617–97 when mice were challenged with 10^3^ CFU (*P* = 0.8485). In this instance only one of five mice had pneumococci detectable in the blood 26 hours post infection (2.1x10^4^ CFU/mL) and none had detectable levels of troponin-I. Note that none of the strains tested showed susceptibility to complement-mediated killing *in vitro* after incubation in whole unfixed human serum for two hours (S3 Fig), suggesting CDC7F:2617–97 was attenuated in some other manner.

### Myocardial damage varies in a strain specific manner

**Fig 2:**
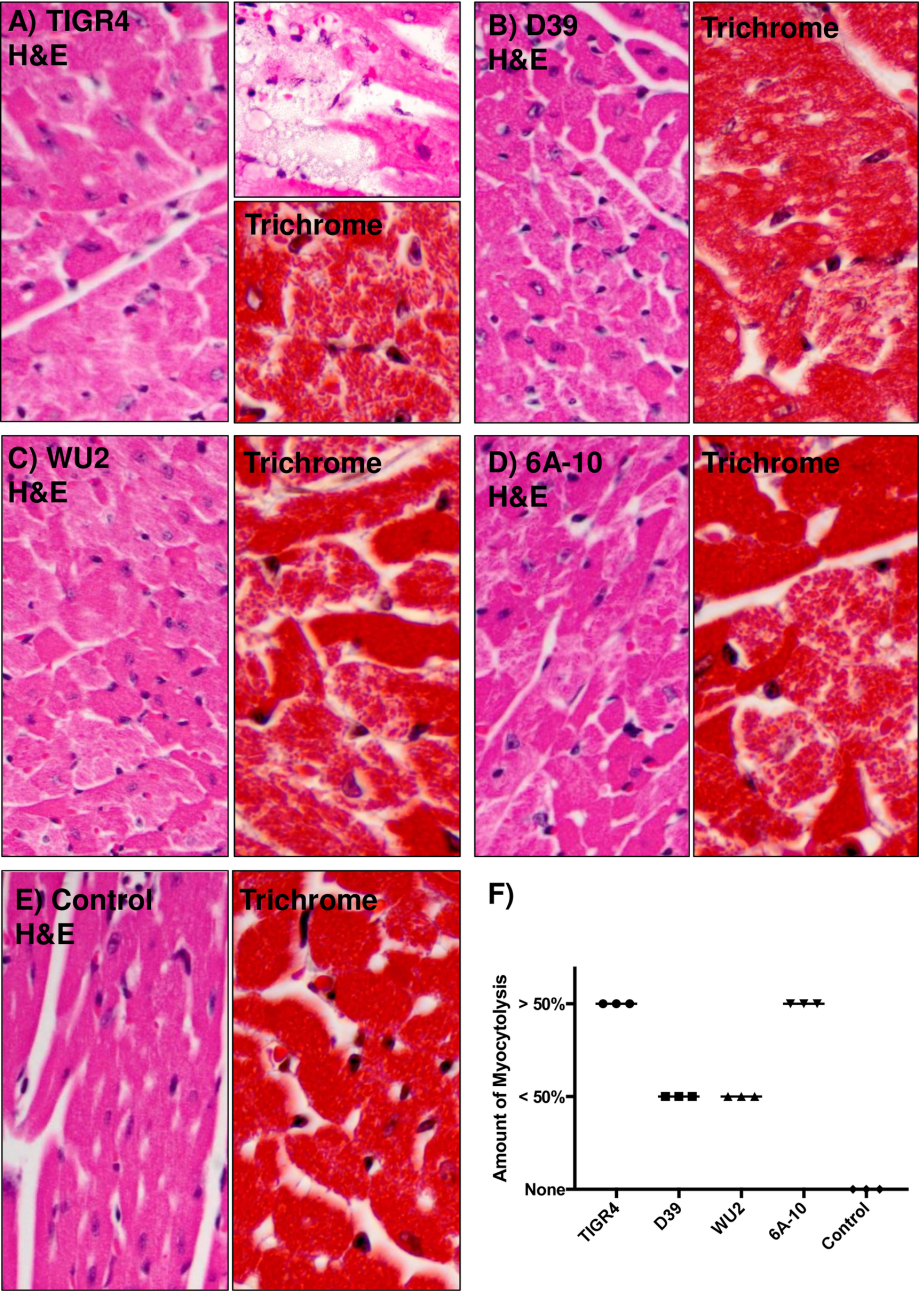
Pneumococcal invasion of the heart and microlesion formation is not required to incite cardiac damage. Representative images of cardiac sections from mice infected with **A)** TIGR4, **B)** D39, **C)** WU2, and **D)** 6A-10, and **E)** an uninfected control. Tissue sections were either stained with H&E or Trichrome. Myocytolysis (i.e. degenerative cardiomyocyte damage visible as lightly stained areas in an otherwise homogenously stained myocardium) and hydropic degeneration of cardiomyocytes is clearly visible across all infected hearts. **F)** H&E stained heart sections from three mice per cohort were scored for extent of myocytolysis as < 50% of myocytes undergoing myocytolysis, > 50% of myocytes undergoing myocytolysis, or no myocytes undergoing myocytolysis.

We next examined H&E and Trichrome stained cardiac sections from D39, WU2, TIGR4, and 6A-10 infected mice for histopathological changes such as presence of bacteria within cardiac tissue, hydropic degeneration of myocytes, and myocytolysis. Strain TIGR4 has previously been shown to cause discrete foci of cardiac damage adjacent to blood vessels [16, 19]. These “cardiac microlesions” are filled with pneumococci and devoid of immune cells. Our results herein recapitulated these published results (Fig 2A). Strain D39 has been reported not to form microlesions but still cause considerable cardiomyopathy [16, 19, 21]. These results were also recapitulated and we observed hydropic degeneration of cells across the myocardium (Fig 2B). Briefly, hydropic degeneration is the swelling of cells due to injury to the membranes affecting ionic transfer; which causes an accumulation of intracellular water. The cardiac damage caused by TIGR4 and D39 was more clearly visible with Trichrome staining, showing discontinuation of cardiomyocyte integrity (Fig 2A and 2B). Diffuse but still overt signs of tissue damage were also detected in cardiac sections from mice infected with WU2 (Fig 2C) and 6A-10 (Fig 2D). This was evidenced by myocytolysis, which is degenerative cardiomyocyte damage visible as lightly stained areas in an otherwise homogenously stained myocardium (Fig 2A–2D) versus controls (Fig 2E). Blinded scoring by a trained pathologist indicated that TIGR4 and 6A-10 consistently resulted in >50% myocytolysis while D39 and WU2 resulted in less severe damage. Importantly, the hearts from uninfected mice showed no myocytolysis (Fig 2F).

Our strains were subsequently tested for the ability to produce the toxin pneumolysin, adhesin CbpA, and H_2_O_2_. These are established pneumococcal virulence determinants [18]. For every single strain tested aside from TIGR4*Δply*, our negative control, a unique 54 kDa band corresponding to the size of pneumolysin could be detected, indicating that all of the serotypes produced this toxin; albeit at different levels. Unexpectedly, levels of pneumolysin correlated negatively with the capability of these strains to cause disease in mice (S4 Fig). We were unable to detect CbpA in WU2, 6A10, and CDC23F:2216–94 samples. Interestingly, unspecified reactivity was seen for AMQ16 when stained for CbpA (S5 Fig). Finally, a general peroxide measurement was taken to assess H_2_O_2_ production by these pneumococci. All strains produced at least 80 μM H_2_O_2_ and no correlation between H_2_O_2_ levels produced and disease severity was observed (S6 Fig). Thus, differences in the production of these virulence determinants do not seem to explain their difference in disease phenotypes.

### Cardiomyocyte death is not always localized with detectable pneumococci

**Fig 3:**
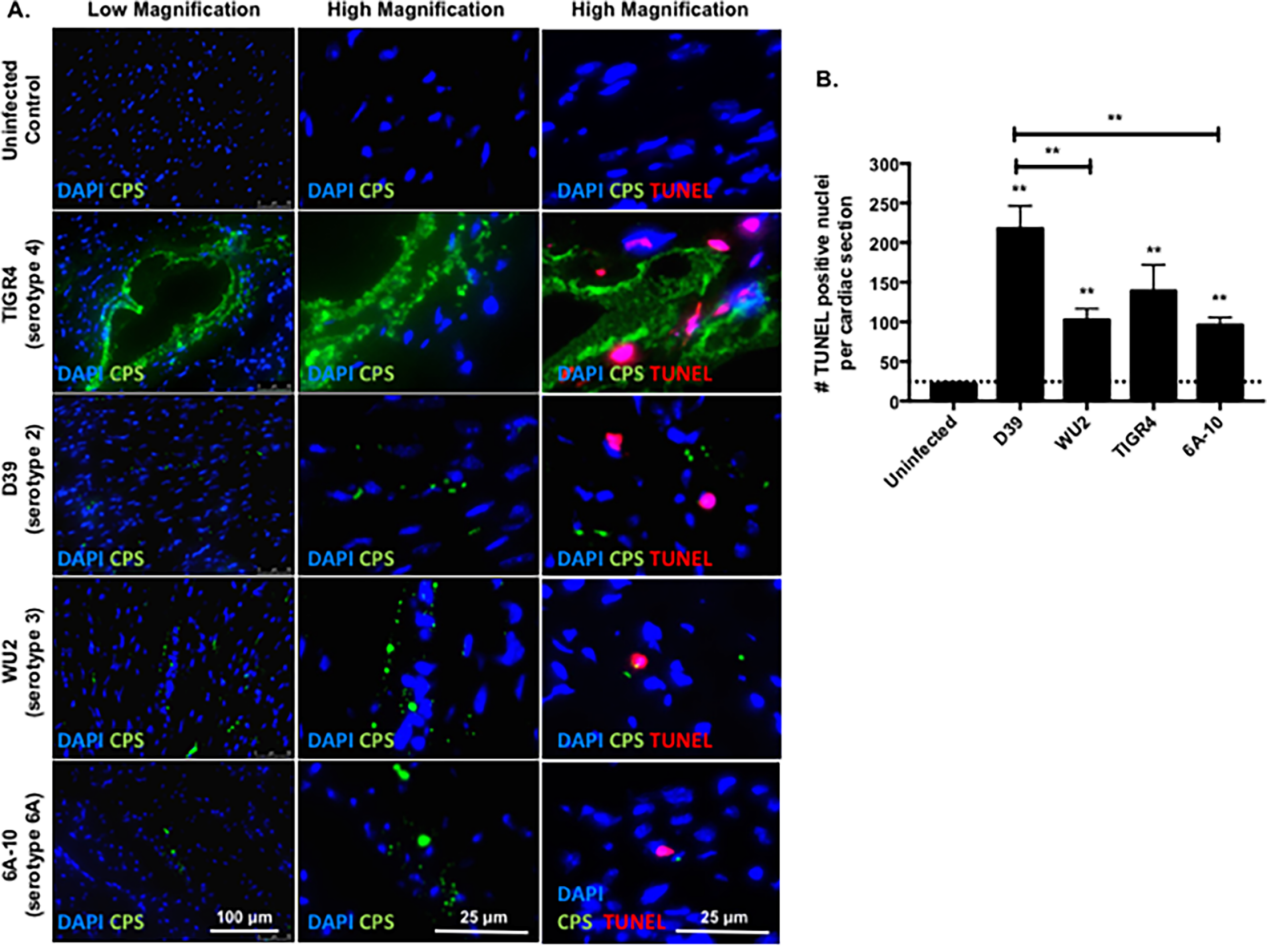
TUNEL staining reveals cardiomyocyte death close to TIGR4, but dispersed damage in hearts from mice challenged with other strains. **A)** Representative low and high magnification immunofluorescent microscopy images of cardiac sections form mice infected with TIGR4, D39, WU2, and 6A-10 pneumococci 30 hours post infection. The heart sections were probed for *S*. *pneumoniae (green)*, nuclei (*blue*, DAPI) and fragmented nuclei (*red*, TUNEL probe). **B)** Enumeration of TUNEL positive nuclei per cardiac section of mice infected with TIGR4, D39, WU2, and 6A-10 pneumococci 30 hours post-infection. For each strain, 4–6 sections were examined. Statistical analyses were performed using a non-parametric one-way ANOVA. *P* value: ** ≤ 0.01, **** < 0.0001; data is represented as mean ± SEM.

Given the stark differences in pathology, we sought to assess cardiomyocyte death in the heart using immunological methods, and further, to determine if the diffuse damage that was observed across the heart was directly associated with the presence of bacteria. TUNEL staining for broken DNA revealed positive nuclei in close proximity to TIGR4 microlesions (Fig 3A). In contrast, hearts of mice infected with strains D39, WU2, and 6A-10 exhibited TUNEL-positive nuclei (dead cells) dispersed across the sections; these were not necessarily adjacent to the bacteria (Fig 3A). Enumeration of the TUNEL-positive nuclei within the infected myocardium revealed that microlesion-incapable strain D39 incited the most cell death, followed by strains TIGR4, WU2, and 6A-10 (Fig 3B).

## Discussion

Severe pneumococcal disease is an established risk factor for adverse cardiac events in hospitalized adults [14, 15]. Direct invasion of the heart by systemically-circulating *S*. *pneumoniae* has been implicated as a cause of cardiac injury that contribute to PACE. This direct cardiotoxicity most likely exacerbates the physiological stresses placed on the heart during severe disease to collectively cause PACE [34]. While previous studies aimed at understanding the basis of cardiac complications post-infection were done using the highly-invasive and commonly used lab strains, serotype 4 strain TIGR4 and serotype 2 strain D39, it was unknown whether other clinical pneumococcal strains could also incite cardiac damage. Herein, using a comprehensive panel of nine *S*. *pneumoniae* clinical isolates belonging to eight different serotypes, we report that cardiac damage is most likely dependent on the ability a clinical isolate to cause high-grade bacteremia and that the pathological attributes of this damage are in turn highly strain dependent.

Our observation that only strains that could cause sustained high-grade bacteremia (D39, WU-2, TIGR4, and 6A-10) were recovered in a meaningful manner from the hearts is consistent with prior work that shows pneumococci gain access to the heart by crossing the cardiac microvasculature [16, 17]. Unclear is if clinical isolates, other than CDC7F:2617–97, that did not cause bacteremia (e.g. AMQ16 or BHN97) would cause heart damage if the animal had been immunosuppressed or infected with a higher-dose and the host defense overwhelmed. Nonetheless, our observation that CDC7F:2617–97 only had slightly higher cardiac involvement, despite a 100-fold increase in the bacterial challenge dose, indicates that the intrinsic virulence of each strain is a critical aspect to causing cardiac damage, perhaps equal as the host defense. This is clinically relevant as pneumococci are highly diverse in their genetic content. The observation that CDC7F:2617–97 was not able to cause disease in experimentally immunocompromised mice suggests attenuation during bacteremia was due to differences in virulence gene expression that would affect nutrient acquisition or due to other host factors. One such possibility is peritoneal, splenic, and cardiac macrophages; although our approach to deplete neutrophils was not precise and resident macrophages and dendritic cells may have also been affected. Importantly, we observed 10,000 bacteria in the heart of one mouse infected with CDC7F:2617–97 that had a blood titer less than 1,000 CFU/mL. This is likely a consequence of clearing the bacteria in the bloodstream whereas those in the heart were capable of sustained replication. This has recently been shown by Ercoli et al. to occur following a transient bacteremia episode, with pneumococci replicating within splenic macrophages [37]. Pneumococci within infected splenic macrophages serve as a source of bacteria during resurgent sepsis.

For those strains that could invade the heart, the type of cardiac damage was found to be strain specific. We observed three general categories of cardiac damage: acute microlesions not associated with immune cells (caused only by TIGR4), infiltrated immune cells at sites where bacteria were present (best exemplified by D39), and cell death at tissue sites where bacteria were not present. Importantly, the amount of cardiac damage as measured by troponin-I, was positively correlated with severity of bacteremia. This, too, is supported by the literature, as it is those individuals with the most severe forms of pneumococcal infection who require admission to the ICU and that are most likely to develop PACE [38]. This has also been seen in non-human primates, where animals with mild pneumonia had the lowest levels of cardiac troponin in serum [39]. Importantly, the specific type of cardiac damage did not seem to be dependent on the number of bacteria that were within the heart suggesting damage was instead due to strain specific effects.

An assessment of CbpA levels and H_2_O_2_ production for our strains found no correlation between these factors and the ability to cause disease nor the type of cardiac damage that was experienced. A negative correlation for pneumolysin production was observed, in particular those that produced the least amount of pneumolysin were those that caused the greatest amount of cardiac damage. This unexpected result can perhaps be explained by the recent report that pneumolysin impairs pneumococcal translocation across vascular endothelial cells [40]. Importantly, we acknowledge that nutrient rich culture media is certainly not representative of *in vivo* conditions. Thus, our studies that examine levels of these determinants need to be interpreted with caution. Future efforts to address this include an examination of virulence gene expression levels *in vivo* and are ongoing.

Importantly, cardiac cytotoxicity was not always observed in cardiac cells adjacent to pneumococci, suggesting released bacterial factors such as pneumolysin circulating within the bloodstream or diffusing away from the pneumococci in the heart might play a role. Prior studies by Alhamdi et al. show that circulating pneumolysin is capable of causing cardiac damage [21]. Alhamdi et al. suggested that all cardiac injury was due to circulating pneumolysin, as they did not detect heart-invaded D39. This was subsequently proven erroneous, as D39 is detected within hearts; however, the concept that pneumolysin in the circulation can cause disseminated heart damage is viable and possibly an explanation for the results that are observed herein for bacteria-independent heart damage. One important consideration is, however, that bacteria are indeed close to the dying cells, but not present within the heart section that was ultimately visualized. One key aspect of this is that with the exception of TIGR4, heart damage was not localized and was spread across the ventricle, presumably affecting heart function as a whole. Note that high-grade bacteremia would allow for sufficient pneumolysin to be released to cause this form of disseminated damage.

In summary, our results show that high-grade bacteremia is requisite for pneumococcal invasion of the heart and cardiac damage. The high-grade bacteremia allows for sufficient levels of cardio-invasion, and perhaps also high levels of noxious products that can kill cells distally to the bacteria. In turn, the specifics of the cardiac damage appear to be highly strain-dependent and not readily explainable by our limited analyses of bacterial virulence determinants *in vitro*. Taken together, this work provides better insight into the type of pneumococcal disease that places individuals at risk for PACE and highlights the previously unknown pneumococcal strain-dependent variability of heart damage that could possibly occur in. It demonstrates that some, but not all, pneumococci are capable of cardiac involvement.

## Supporting information

S1 Table Isolation source of isolates used in this study. (DOCX)

S1 Fig Immunofluorescence staining of hearts from mice infected with different strains of *S*. *pneumoniae*. (PDF)

S2 Fig Mice given high-doses of *S*. *pneumoniae* CDC7F:2617–97 did not become bacteremic. (PDF)

S3 Fig *S*. *pneumoniae* survives in human serum. (PDF)

S4 Fig All nine *S*. *pneumoniae* strains express pneumolysin. Pneumolysin production negatively correlates with ability to cause disease.
(PDF)

S5 Fig Expression of CbpA varies. (PDF)

S6 Fig Production of H_2_O_2_ varies by strain and does not correlate with ability to cause disease. (PDF)

S1 File Supporting information: Bacteria in the heart. (XLSX)

S2 File Supporting information: Troponin. (XLS)

S3 File Supporting information: Serum survival. (XLSX)

S4 File Supporting information: H_2_O_2_ assay. (XLSX)

S5 File Supporting information: Bacterial burden over time. (XLSX)

S6 File Supporting information: ARRIVE checklist. (PDF)
