# Supplementary material for: Severity and properties of cardiac damage caused by Streptococcus pneumoniae are strain dependent
Source: PLoS One. 2018 Sep 14;13(9):e0204032. doi: 10.1371/journal.pone.0204032 (PMC6138390; doi:10.1371/journal.pone.0204032)
Supplement: S1 Table — (DOCX) [file pone.0204032.s001.docx]

| **Strain** | **Source information** | |
| --- | --- | --- |
| D39 | Clinical isolate (year 1916) used in Avery et al.’s famous DNA experiments. | |
| WU2 | Human isolate obtained courtesy of Mr. John Courtney, Washington University School of Medicine. This isolate had been maintained over a period of 15 years by annual passage through mice. After three additional mouse passages, strain WU1 was established from a smooth colony. From a subsequent mouse passage of WU1, a more virulent subline, WU2, was isolated. Two additional mouse passages have not increased the virulence of WU2. | |
| TIGR4 | Isolated from the blood of a 30-year-old male patient from Norway | |
| AMQ16 | Isolated in Mozambique | |
| 6A-10 | Isolated from nasopharyngeal swabs of healthy carriers collected at The University of Texas Southwestern Medical Center in Dallas County, Texas. | |
| CDC7F:2617-97 | Invasive pneumococcal disease isolate |  |
| EF3030 | Clinical otitis media isolate |  |
| BHN97 | Isolated from a healthy carrier | |
| CDC23F:2216-94 | Invasive pneumococcal disease isolate | |

**S1 Table**
