# Supplementary figures and images for: Severity and properties of cardiac damage caused by Streptococcus pneumoniae are strain dependent
Source: PLoS One. 2018 Sep 14;13(9):e0204032. doi: 10.1371/journal.pone.0204032 (PMC6138390; doi:10.1371/journal.pone.0204032)

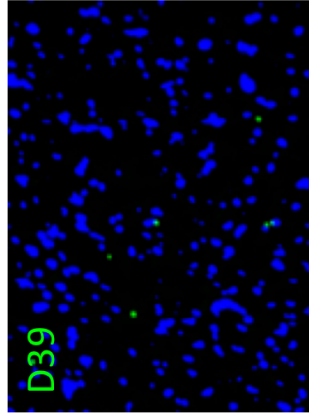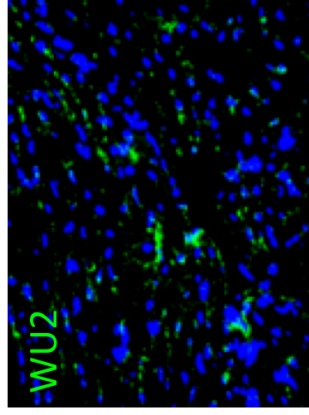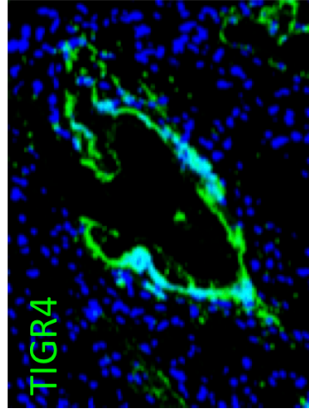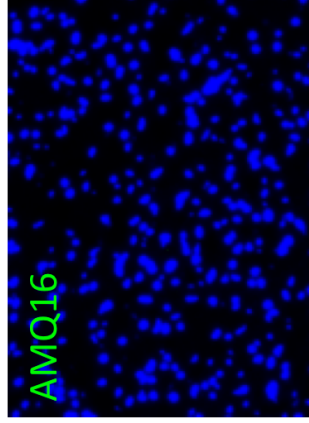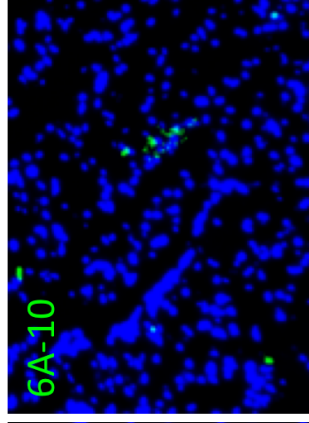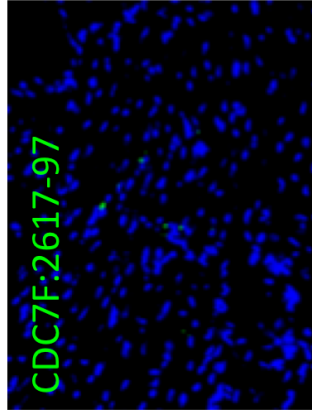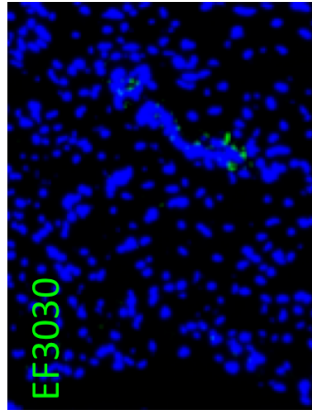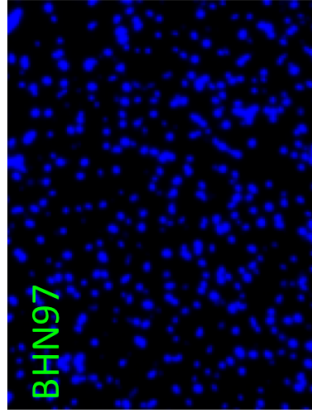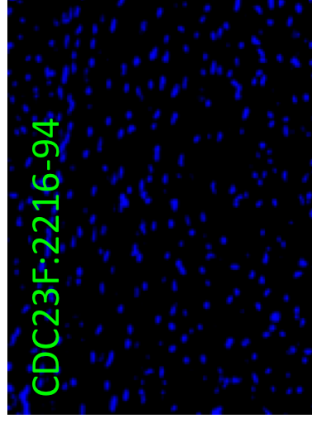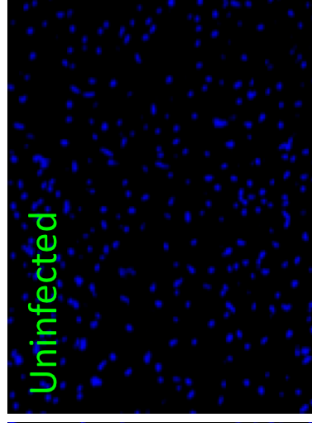

Supplement: S1 Fig — (PDF) [file pone.0204032.s002.pdf]

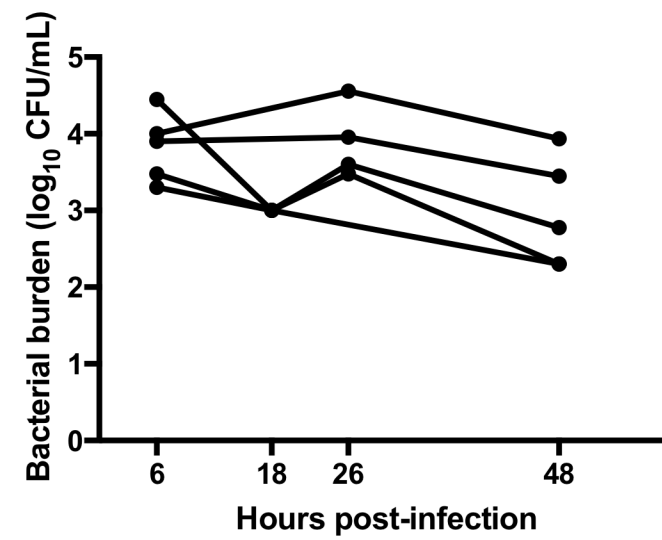

Supplement: S2 Fig — (PDF) [file pone.0204032.s003.pdf]

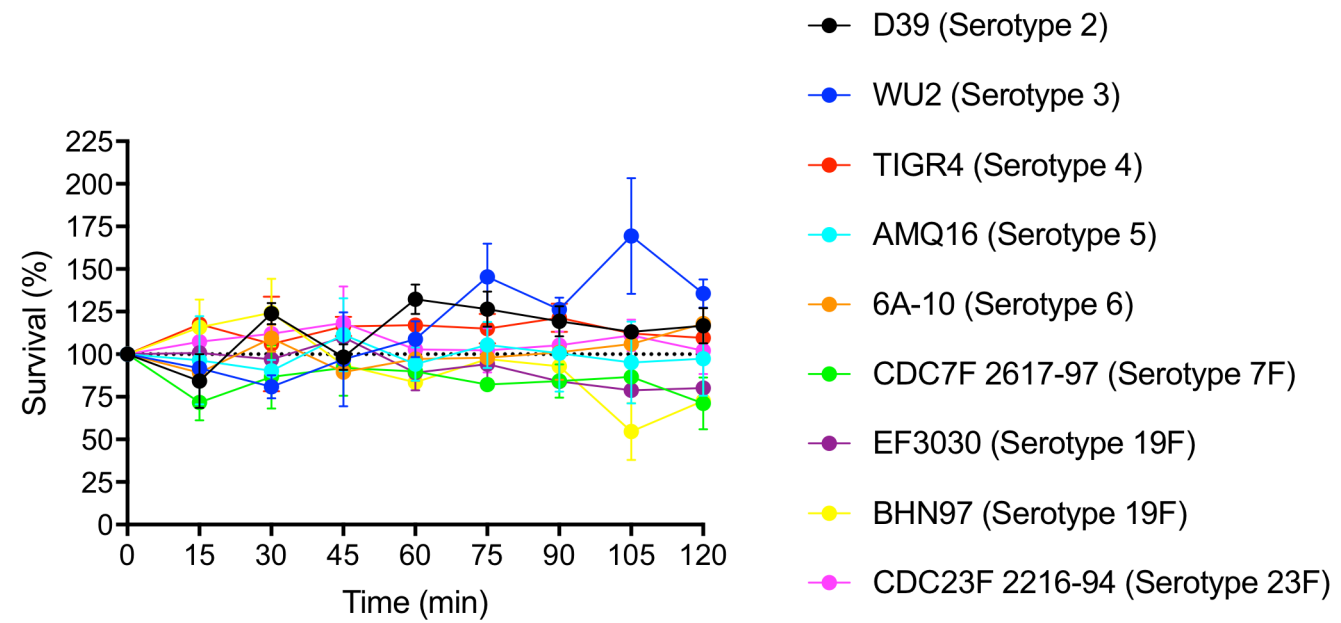

Supplement: S3 Fig — (PDF) [file pone.0204032.s004.pdf]

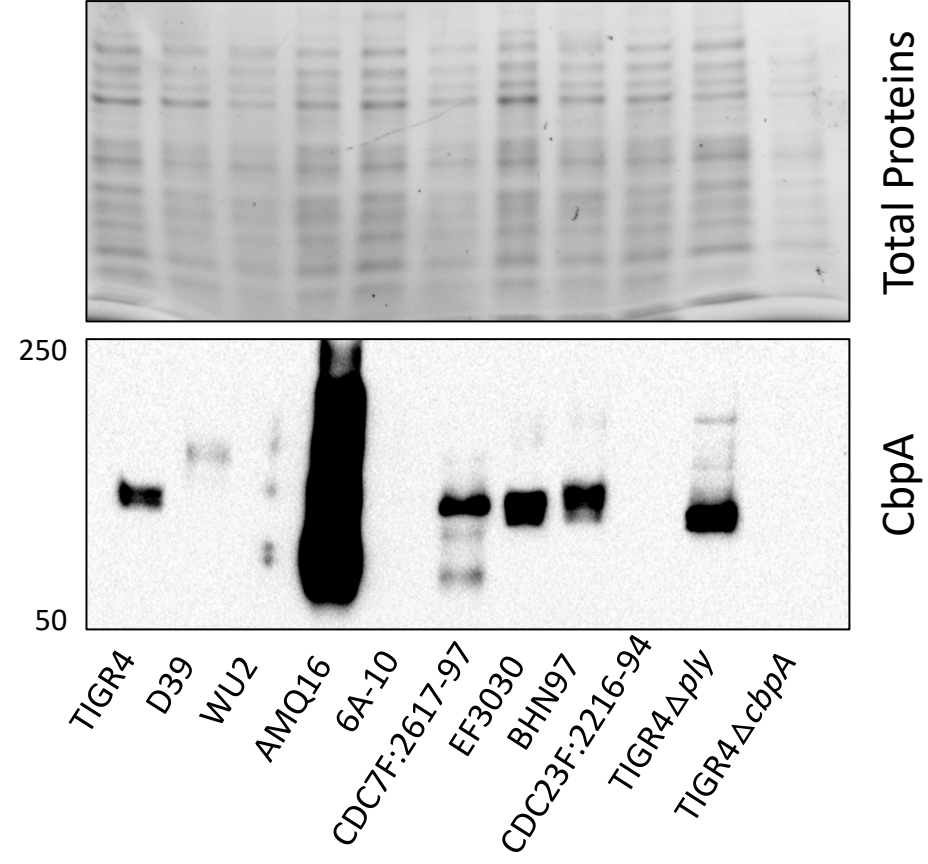

Supplement: S5 Fig — (PDF) [file pone.0204032.s006.pdf]

**A****Raw values**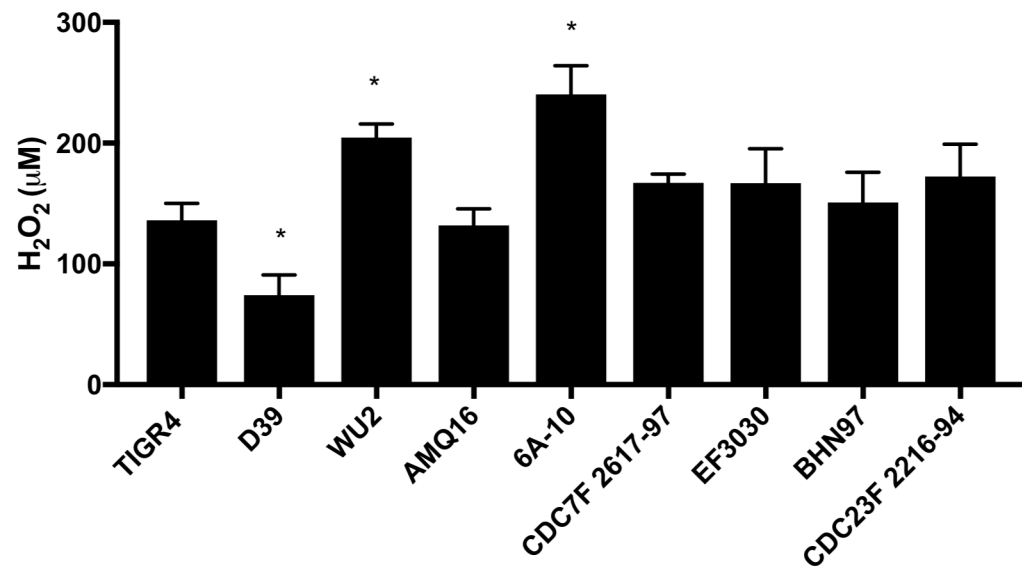**B****Normalized values**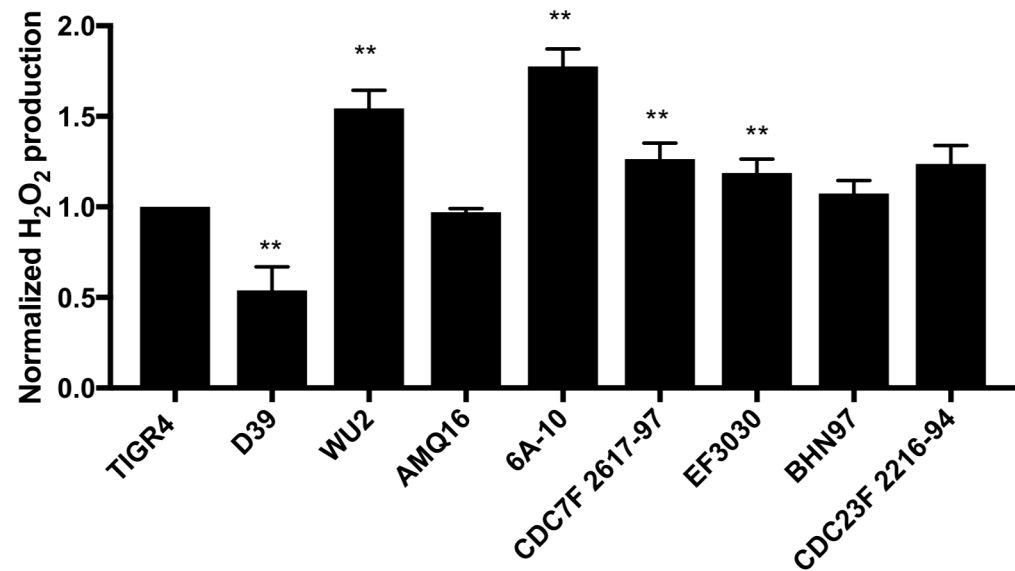**C**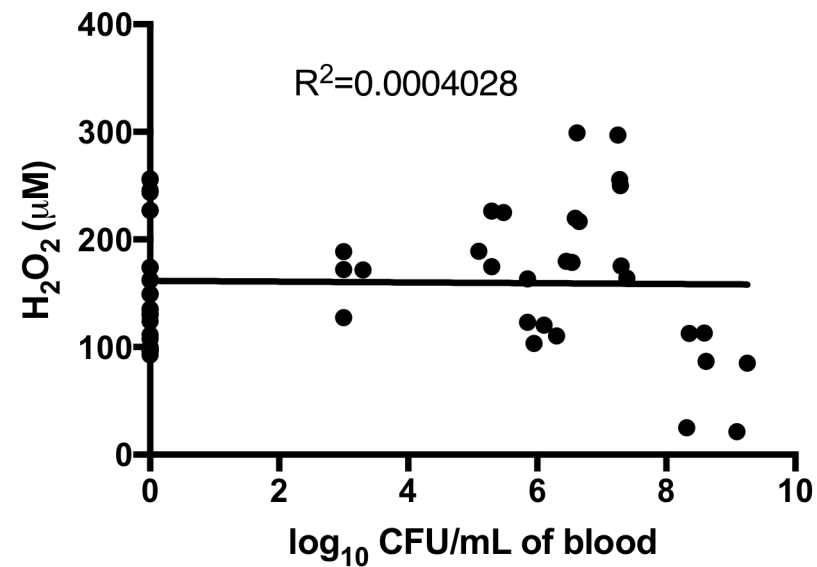

Supplement: S6 Fig — (PDF) [file pone.0204032.s007.pdf]
