# Supplementary material for: Severity and properties of cardiac damage caused by Streptococcus pneumoniae are strain dependent
Source: PLoS One. 2018 Sep 14;13(9):e0204032. doi: 10.1371/journal.pone.0204032 (PMC6138390; doi:10.1371/journal.pone.0204032)
Supplement: S4 Fig — Pneumolysin production negatively correlates with ability to cause disease. (PDF) [file pone.0204032.s005.pdf]

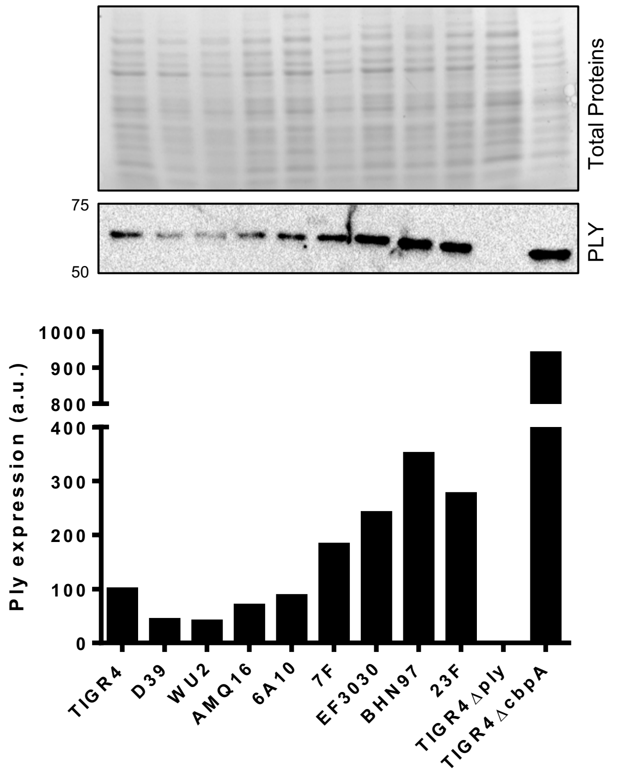
Total Proteins
75
50
PLY
Ply expression (a.u.)
1000
900
800
400
300
200
100
0
TIGR4
D39
WU2
AMQ16
6A10
7F
EF3030
BHN97
23F
TIGR4△ply
TIGR4△cbpA

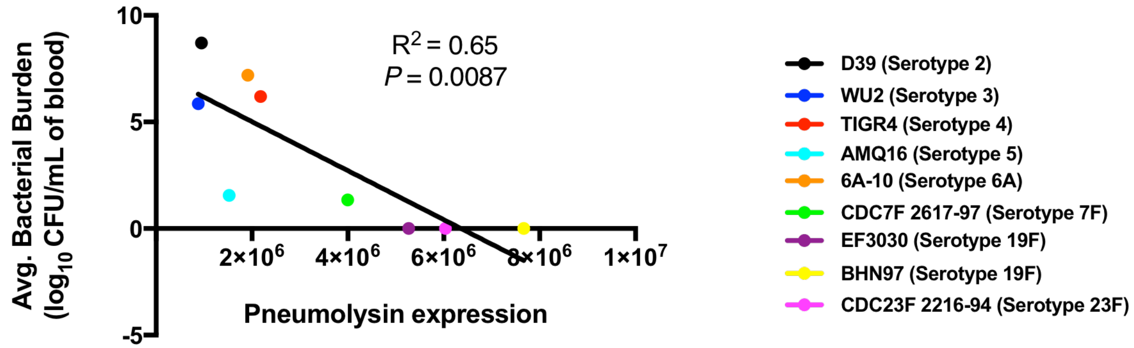
Avg. Bacterial Burden
(log_{10} CFU/mL of blood)
10
5
0
-5
$R^2 = 0.65$
$P = 0.0087$
2×10^6
4×10^6
6×10^6
8×10^6
1×10^7
Pneumolysin expression
D39 (Serotype 2)
WU2 (Serotype 3)
TIGR4 (Serotype 4)
AMQ16 (Serotype 5)
6A-10 (Serotype 6A)
CDC7F 2617-97 (Serotype 7F)
EF3030 (Serotype 19F)
BHN97 (Serotype 19F)
CDC23F 2216-94 (Serotype 23F)
